# One-step process of hydrothermal and alkaline treatment of wheat straw for improving the enzymatic saccharification

**DOI:** 10.1186/s13068-018-1140-x

**Published:** 2018-05-12

**Authors:** Shaolong Sun, Lidan Zhang, Fang Liu, Xiaolin Fan, Run-Cang Sun

**Affiliations:** 1 College of Natural Resources and Environment, South China Agricultural University, Guangzhou, 510642 Guangdong China; 2 Beijing Key Laboratory of Lignocellulosic Chemistry, Beijing Forestry University, Beijing, 100083 China

**Keywords:** One-step process, Hydrothermal and alkaline treatment, Enzymatic saccharification, Wheat straw

## Abstract

**Background:**

To increase the production of bioethanol, a two-step process based on hydrothermal and dilute alkaline treatment was applied to reduce the natural resistance of biomass. However, the process required a large amount of water and a long operation time due to the solid/liquid separation before the alkaline treatment, which led to decrease the pure economic profit for production of bioethanol. Therefore, four one-step processes based on order of hydrothermal and alkaline treatment have been developed to enhance concentration of glucose of wheat straw by enzymatic saccharification. The aim of the present study was to systematically evaluated effect for different one-step processes by analyzing the physicochemical properties (composition, structural change, crystallinity, surface morphology, and BET surface area) and enzymatic saccharification of the treated substrates.

**Results:**

In this study, hemicelluloses and lignins were removed from wheat straw and the morphologic structures were destroyed to various extents during the four one-step processes, which were favorable for cellulase absorption on cellulose. A positive correlation was also observed between the crystallinity and enzymatic saccharification rate of the substrate under the conditions given. The surface area of the substrate was positively related to the concentration of glucose in this study. As compared to the control (3.0 g/L) and treated substrates (11.2–14.6 g/L) obtained by the other three one-step processes, the substrate treated by one-step process based on successively hydrothermal and alkaline treatment had a maximum glucose concentration of 18.6 g/L, which was due to the high cellulose concentration and surface area for the substrate, accompanying with removal of large amounts of lignins and hemicelluloses.

**Conclusions:**

The present study demonstrated that the order of hydrothermal and alkaline treatment had significant effects on the physicochemical properties and enzymatic saccharification of wheat straw. The one-step process based on successively hydrothermal and alkaline treatment is a simple operating and economical feasible method for the production of glucose, which will be further converted into bioethanol.

## Background

Lignocelluloses, whether as agricultural biomass or forestry biomass, provide a unique energy resource for sustainable production of bioethanol [1–3]. Among them, wheat straw is considered as one of the most potential renewable non-woody materials for production of bioethanol in biorefinery industry, since high productivity depends on the specific wheat varieties, geographical distribution, and climatic conditions [4]. Unfortunately, although the energy conversion of wheat straw into bioethanol has momentous economical and market potential, cell wall of wheat straw naturally resists cellulase attack due to many complex physicochemical factors, such as the dense structure of the cell wall, cellulose crystallinity, accessible surface, and the existence of lignin and hemicelluloses [5]. Thus, an effective pretreatment approach must be performed prior to the enzymatic saccharification to overcome the recalcitrance of cell wall of wheat straw and improve the cellulase accessibility on cellulose in the pretreated substrates [6].

Numerous pretreatment methods have been developed to reduce recalcitrance and enhance enzymatic saccharification of wheat straw, such as hydrothermal, alkaline, steam explosion, and organosolv pretreatments [7–10]. Among them, hydrothermal treatment resulted in solubilization of hemicelluloses and redistribution of lignins, whereas alkaline treatment could swell plant cell wall as well as remove hemicelluloses and lignins from wheat straw, which in turn contribute to the reduction of cell wall recalcitrance. However, hydrothermal treatment alone or alkaline treatment alone had limited for improvement of the enzymatic saccharification efficiency of cellulose of wheat straw and could not recycle large amounts of lignins. Thus, a combination of hydrothermal and alkaline treatment was studied and considered to be a promising integrated technology that could improve sugar release performance for wheat straw [11]. Meanwhile, the same combination treatment under different conditions has also been proposed to enhance the availability for the application of three major components (cellulose, hemicelluloses, and lignin) of sweet sorghum as biochemical and biofuels (xylooligosaccharide, high-purity lignin, and cellulose-rich substrate to produce glucose for ethanol production) [12]. However, due to the solid/liquid separation before the alkaline treatment during the combined treatment process, the combined treatment requires a large amount of water and a long operation time, meanwhile resulting in the hydrothermal and alkaline treatment being discontinuous, which defined as a two-step process. For the industrial process, it will decrease the pure economic profit for the application of biomass, since water consumption as well as operating and environmental costs increased.

Based on the above disadvantages, a combined treatment with simple and feasible method based on order of hydrothermal (170 °C, 30 min) and alkaline treatment (2% NaOH, 90 °C, 120 min) have been proposed for the application of three major components (cellulose, hemicelluloses, and lignin) of wheat straw as biochemical and biofuels. During the combined treatment, there is no requirement of solid/liquid separation before the alkaline treatment. Therefore, it is defined as a one-step process of the continuous treatment. In the present study, the enzymatic saccharification of the substrates obtained from one-step process based on the order of hydrothermal and alkaline treatment was thoroughly investigated for production of fermentable glucose. All substrates obtained were analyzed by chemical composition, solid-state cross-polarization/magic angle spinning ^13^C nuclear magnetic resonance (CP/MAS ^13^C NMR), X-ray diffraction (XRD), scanning electron microscopy (SEM), and Brunauer–Emmett–Teller (BET) surface area, and their concentrations of glucose and xylose by enzymatic saccharification were also measured. Simultaneously, large amounts of lignins were obtained by one-step process. The dissociation mechanism and structural transformations of the lignins will be thoroughly investigated in another forthcoming article. These results will provide some important information for the commercial potential of wheat straw on industrial production of bioethanol in future biorefinery.

## Results and discussion

### Compositional analysis

**Fig. 1:**
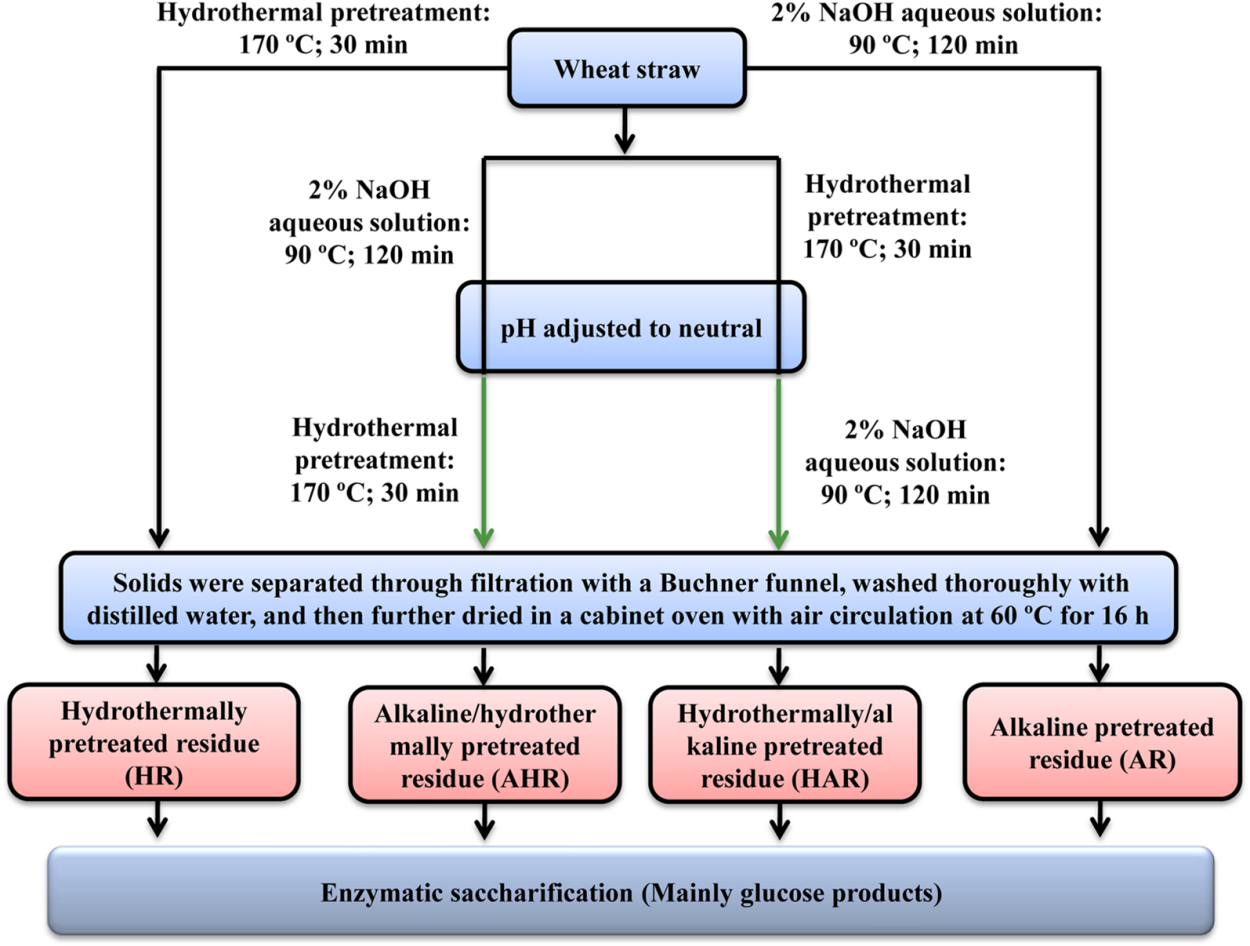
Schematic illustration of one-step process based on order of hydrothermal and alkaline treatment

To obtain higher concentrations of glucose and xylose converted from wheat straw for production of bioethanol, hemicelluloses and lignins in the control substrate must be extensively removed prior to enzymatic saccharification. Therefore, the four one-step processes based on order of hydrothermal and alkaline treatment were developed to remove the hemicelluloses and lignins from wheat straw for the subsequent production of bioethanol and the scheme is illustrated in Fig. 1. Aforementioned, the four one-step processes were named HP, AP, AHP, and HAP, respectively. Meanwhile, at the end of the four reactions, the residues obtained from the corresponding processes were named HR, AR, AHR, and HAR, respectively (Fig. 1). The composition of the control substrate, which was determined by National Renewable Energy Laboratory’s (NREL) standard analytical method, was 42.8% glucan, 24.4% xylan, and 21.1% lignin (20.1% Klason lignin and 1.0% acid-soluble lignin) [13].

It is well known that the prime target of pretreatment is to overcome the recalcitrance of cell wall of lignocelluloses, since they could inhibit enzymatic saccharification of cellulose [14, 15]. In the present study, hydrothermal treatment was applied to degrade and remove xylan, disrupt the structure of cell wall, thus increasing the enzyme accessibility of the substrates [16]. The main degraded components during the hydrothermal treatment were xylan, which is manifested in Table 1. After hydrothermal treatment alone, the content of xylan (10.1%) in the HR obviously decreased as compared to the control substrate (24.4%). In addition, it has been reported that alkaline treatment is a promising technology for the effective removal of hemicelluloses and lignins, which can dramatically enhance enzymatic saccharification of the substrates [17]. Therefore, sodium hydroxide was also used alone or combined with hydrothermal treatment to remove hemicelluloses and lignins from wheat straw to improve enzymatic saccharification of cellulose. As can be seen, after alkaline treatment alone, the contents of hemicelluloses (19.7%) and Klason lignin (9.7%) of the AR remarkably reduced as compared to the control substrate, especially for lignins. Unfortunately, although hydrothermal alone and alkaline alone treatment had significant economical and market potential for removing hemicelluloses and lignin, one-step process based on successively alkaline and hydrothermal treatment had not achieve the anticipated effect. As can been seen from Table 1, the AHR contained relatively higher xylan (14.5%) as compared to the HR and Klason lignin (18.9%) as compared to the AR. Specially, the HAR contained relatively higher cellulose (78.6%), but lower xylan (7.8%) and Klason lignin (5.4%) as compared to the HR, AR, and AHR, suggesting that the HAP was more effective to degrade hemicelluloses and lignin than the other three one-step processes. In other words, successively hydrothermal and alkaline treatment was more significant on the removal of hemicelluloses and lignin from the wheat straw during the one-step process based on order of hydrothermal and alkaline treatment.

**Table 1:** Chemical compositions (w/w, %) of the substrates obtained from the four one-step processes

Sample	Glucan	Xylan	KL^a^	ASL^b^
Control	42.8	24.4	20.1	1.0
HR^c^	60.1	10.1	25.5	0.7
AR	63.7	19.7	9.7	0.5
AHR	57.6	14.5	18.9	0.4
HAR	78.6	7.8	5.4	0.7

^a^*KL* Klason lignin (i.e. acid insoluble lignin)

^b^*ASL* acid soluble lignin

^c^*HR* the residue obtained from hydrothermal treatment alone

*AR* the residue obtained from alkaline treatment alone

*AHR* the residue obtained from one-step process based on successively alkaline and hydrothermal treatment

*HAR* the residue obtained from one-step process based on successively hydrothermal and alkaline treatment

### Enzymatic saccharification

**Fig. 2:**
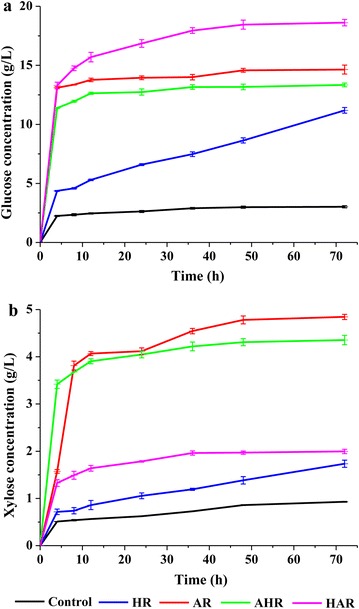
Concentrations of glucose (**a**) and xylose (**b**) of the substrates obtained from the four one-step processes by enzymatic saccharification. *HR* the residue obtained from hydrothermal treatment alone, *AR* the residue obtained from alkaline treatment alone, *AHR* the residue obtained from one-step process based on successively alkaline and hydrothermal treatment, *HAR* the residue obtained from one-step process based on successively hydrothermal and alkaline treatment

The amenability of the treated substrates to enhance conversion of cellulose and xylan was demonstrated by enzymatic saccharification and the results are shown in Fig. 2. One-step process based on order of hydrothermal and alkaline treatment could effectively improve the enzymatic saccharification of the wheat straw in this study. The concentrations of glucose and xylose were just 3.0 and 0.9 g/L for the control substrate after 72 h enzymatic saccharification. After hydrothermal treatment alone, these two values enhanced significantly and reached to 11.2 and 1.7 g/L, respectively.By contrast, these two values further increased to 14.6 and 4.8 g/L after alkaline treatment alone. These data showed that alkaline treatment alone was more effective to enhance concentrations of glucose and xylose by enzymatic saccharification than hydrothermal treatment alone, which was probably attributable to the content of lignin in AR lower than that of HR.

Based on the above results, two novel processes were proposed to enhance concentrations of glucose and xylose by enzymatic saccharification. As can be seen, the concentrations of glucose and xylose of the AHR obtained from the AHP based on successively alkaline and hydrothermal treatment were 13.3 and 4.4 g/L for the AHR, whereas the concentrations of glucose and xylose of the HAR obtained from the HAP based on successively hydrothermal and alkaline treatment reached to 18.6 and 2.0 g/L, respectively. In all of the prepared substrates (control, HR, AR, AHR, and HAR), the highest concentration of glucose was obtained from the HAR, which was probably related to the removal of a large amount of lignin and hemicelluloses during the HAP. Hence, the increasing removal amount of lignins and xylans was positively correlated with an increasing enzymatic saccharification of cellulose. In addition, the highest concentration of xylose was achieved from the AR. This result was mainly attributed to that AR had a relatively higher content of xylan, but lower content of Klason lignin. In short, successively hydrothermal and alkaline treatment could significantly improve the concentration of glucose of the substrate, while alkaline treatment alone was more significant on the enhancement of xylose of the substrate.

### CP/MAS ^13^C-NMR spectral analysis

**Fig. 3:**
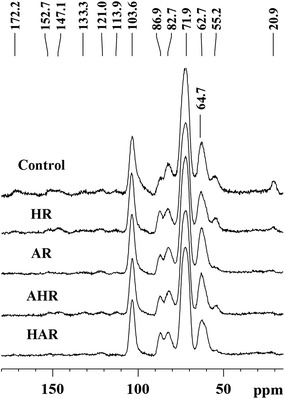
CP/MAS ^13^C NMR spectra of the substrates obtained from the four one-step processes

Solid-state CP/MAS ^13^C-NMR technique can provide detailed information for structural changes of the substrates obtained from four different processes (Fig. 3). All significant signals are distributed in the region of 60.0 − 110.0 ppm and derived from the carbons of polysaccharides, mainly cellulose. The region between 86.0 and 92.0 ppm is originated from the crystalline region of cellulose, whereas amorphous regions of cellulose are distributed in the region of 80.0 − 86.0 ppm. Among them, the overlapping three-signal from C-2, C-3, and C-5 of cellulose showed a strong signal at 71.9 ppm. The signals at 86.9 and 64.7 ppm correspond to the C-4 and C-6 of the crystalline cellulose, while the signals at 82.7 and 62.7 ppm originate from the C-4 and C-6 of the amorphous cellulose, respectively. Two intensive signals at 172.2 (C=O conjugates) and 20.9 (C–O) ppm were observed clearly in the spectrum of control substrate and derived from the acetyl group of hemicelluloses. The signals of lignins the substrates were found at 152.7, 147.1, 133.3, 121.0, 113.9, and 55.2 ppm, which are assigned to S_3,5_ (etherified), S_3,5_ (non-etherified), S_1_/S_4_ (non-etherified), G_6_, G_5_, and OCH_3_, respectively [18]. It was noted that the spectra of the substrates presented different patterns after four different processes. For example, the intensities of the signals of hemicelluloses in the treated substrates reduced to some extent as compared to that of the control substrate, indicating that the removal of hemicelluloses occurred during the four one-step processes, which resulted in rise of the concentration of glucose in the treated substrates. As expected, the intensities of the signals of lignins also appeared similar varying tendency in the corresponding substrates, i.e., the intensities of the signals of lignins dropped after the four one-step processes, especially during the AP and HAP. It is interesting to note that the concentration of glucose of the substrates was closely related to the lignins’ content. As can be seen from Table 1 and Fig. 2, the HAR with the lowest contents of lignins (5.4%) and hemicelluloses (7.8%) exhibited the highest concentration of glucose as compared with the other prepared substrates (control, HR, AR, and AHR). The concentration of glucose by enzymatic saccharification was mainly influenced by the contents of lignins and hemicelluloses in the substrates. Similar result was observed in the previous literature [19, 20]. These factors were also implied that the reduction of contents of lignins and/or hemicelluloses favored the enzymatic saccharification of the substrates.

### Crystallinity of substrate

**Fig. 4:**
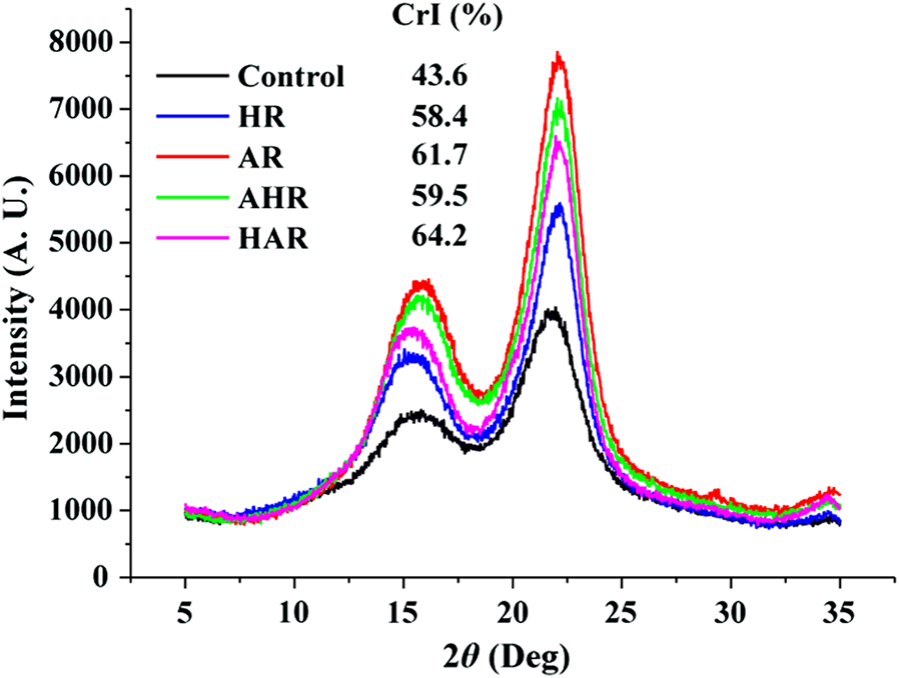
XRD spectra of the substrates obtained from the four one-step processes

Among the diverse physicochemical characteristics effecting enzymatic saccharification of substrates, the crystallinity of cellulose is regarded as one of the main features of substrate that influence saccharification kinetics [21–23]. In this study, XRD analysis technique was applied to determine the crystallinity indexes (CrIs) of the substrates. Figure 4 shows the XRD patterns and CrIs of the control, HR, AR, AHR, and HAR. The peak of amorphous cellulose appeared at 18.5°, and the peaks at 16.5° and 22.5° were attributed to the crystalline cellulose. As compared with CrI of the control substrate (43.6%), the CrIs of the HR and AR, which were subjected to the hydrothermal alone and alkaline alone treatment, increased by 14.8 and 18.1%, respectively. This was attributed to the fact that the removal of amorphous lignins and hemicelluloses during the HP and HR was also revealed by the results of compositional analysis of the substrates [24]. Meanwhile, it was found that the removal total amounts of amorphous lignins and hemicelluloses during the HP were lower than that during the AP, resulting in a higher content of cellulose in the AR with a higher CrI as compared to that of the HR. Similar results could be observed for the wheat straw by different processing methods; that is, CrI increased with removal of amorphous lignins and hemicelluloses [25, 26]. As expected, in the current study, after one-step process based on successively hydrothermal and alkaline treatment, the CrI of the HAR obtained was obvious higher than those of the AR and HR. However, after one-step process based on successively alkaline and hydrothermal treatment, the CrI of the AHR obtained was obvious higher than that of the HR, whereas lower than that of the AR. The difference might be attributed to the various process orders based on alkaline and hydrothermal treatment.

According to the CrIs data of the substrates combined with the results of enzymatic saccharification, it was found that the CrI was positively related to the enzymatic saccharification in this study. In fact, the correlation between CrI and enzymatic saccharification has been extensively investigated [27]. A positive correlation was also observed between the CrI and enzymatic saccharification rate of the substrate under no transformations of XRD patterns conditions. However, the CrI was reduced during some specific pretreatments, which induced the transformations of XRD patterns of the substrate, favoring the cellulose II from cellulose I or formation of amorphous cellulose. Under these pretreatments, the obtained substrates were more readily saccharification than cellulose I, such as during phosphoric acid pretreatment and ionic liquid pretreatment [28, 29].

### Surface morphology

**Fig. 5:**
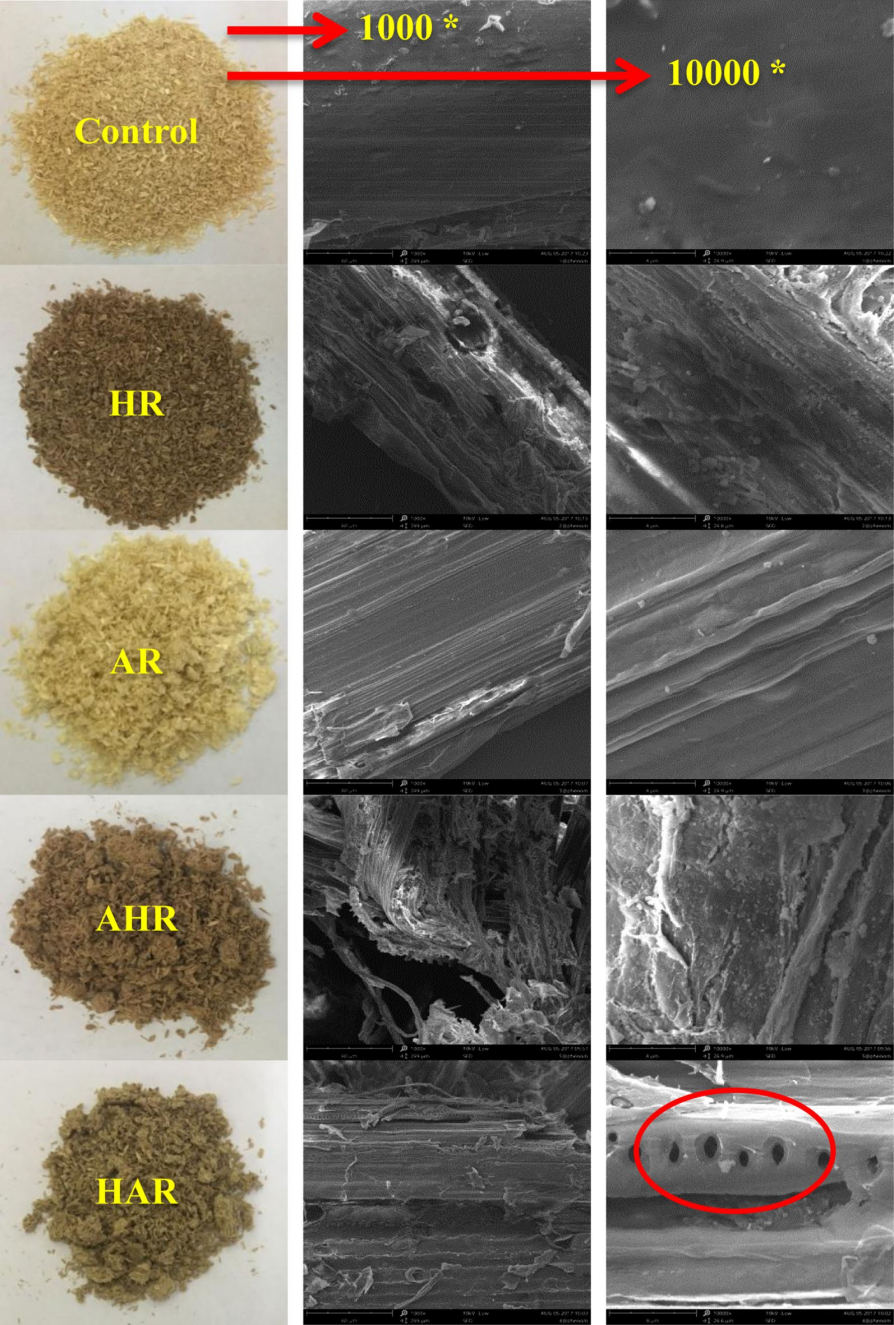
SEM images of the substrates obtained from the four one-step processes (magnifications are 1000 and 10,000)

Various processes based on order of hydrothermal and alkaline treatment could significantly alter the surface morphology of wheat straw, which also influenced the enzymatic saccharification of the substrates. To investigate the surface morphology changes caused by the four one-step processes, SEM images of the control, HR, AR, AHR, and HAR were observed at two magnifications (1000 and 10,000) (Fig. 5). The control substrate exhibited a rigid surface morphology, which could hinder the accessibility of cellulose to cellulases [30]. By contrast, there were distinct differences in HR and AR obtained from the HP and AP as compared to that of the control substrate, especially for HR. Specifically, the surface of the HR was broken and appeared cracks and small particle-sized debris, whereas the surface of the AR exhibited smooth and fiber bundles. The results agreed well with the previous findings in which different lignocellulosic materials (barley straw, rice straw, and eucalyptus bark) under various hydrothermal and alkaline conditions [31–33]. These changes were closely related to the process orders and conditions since the difference of diverse mechanism on removal of hemicelluloses and lignin from the control substrate. In brief, hydrothermal alone and alkaline alone treatment broke the original dense structure of the control substrate and facilitated the subsequent enzymatic saccharification, which resulted in enhancing concentrations of glucose and xylose from the HR and AR.

As compared with the AR, the surface of the AHR was broken into separated fibers or fiber bundles and emerged a large amount of particle-sized debris, which resulted in reduced concentrations of glucose and xylose from the AHR by enzymatic saccharification, probably related to the partial cellulase adsorption on particle-sized debris of the substrate [34–36]. By contrast, the surface of the HAR became smooth as compared to the HR. This was possible that most of lignins and residual hemicelluloses were removed by the alkali during the HAP. Meanwhile, the surface of the HAR appeared some porous structures. All these changes released large amounts of reactive sites on the fiber surface in the HAR, thereby enhancing the accessibility of enzymes and then enzymatic saccharification. Therefore, the novel process based on successively hydrothermal and alkaline treatment is a promising technique for improving cellulose saccharification of wheat straw.

### BET surface area

Four one-step processes based on hydrothermal and alkaline treatment also led to the changes of BET surface areas of the substrates (Table 2). It is recognized that the increment of surface area enhanced accessibility of cellulase to cellulose, thus improving the enzymatic saccharification of the substrate [37–39]. In the present study, the substrates obtained from the four processes showed relatively higher surface areas (7.7 − 12.9 m^2^/g) as compared to that of the control substrate (6.4 m^2^/g). This increased trend was well correlated with breakdown degree of the microstructures of the substrates. As expected, the removal of most lignin and hemicelluloses during the HAP incurred a higher surface area for the HAR as compared with the HR and AR. In fact, the substrates with higher surface areas were beneficial to promote the contact between cellulase and cellulose in the substrates, which resulted in enhanced concentration of glucose by enzymatic saccharification. In other words, the surface area of the substrate was positively related to the concentration of glucose under the conditions given. For example, the control substrate with the lowest surface areas (6.4 m^2^/g) had the lowest concentration of glucose (3.0 g/L), whereas the HAR with the highest surface areas (12.9 m^2^/g) showed the highest concentration of glucose (18.6 g/L). Similarly, the same result was found in a previous study, in which enzymatic saccharification was positive correlation to the available surface area [12]. Thus, size-reduction by multiple refining was indispensable for the improvement of enzymatic saccharification of substrate in future biorefinery industry.

**Table 2:** BET surface area (m^2^/g) the substrates obtained from the four one-step processes

	Control	HR	AR	AHR	HAR
BET surface area	6.4	7.7	10.4	9.3	12.9

## Conclusions

Four one-step processes based on order of hydrothermal and alkaline treatment were applied to enhance concentration of glucose from wheat straw by enzymatic saccharification. After different processes, hemicelluloses and lignins were removed from wheat straw and the morphologic structures were destroyed to various extents, which were favorable for cellulase absorption on cellulose. A positive correlation was also observed between the crystallinity and enzymatic saccharification rate of the substrate under the conditions given. The surface area of the substrate was positively related to the concentration of glucose in this study. As compared to the control (3.0 g/L) and treated substrates (11.2–14.6 g/L) obtained by the other three one-step processes, the substrate treated by one-step process based on successively hydrothermal and alkaline treatment showed a maximum glucose concentration of 18.6 g/L, which was due to the high cellulose concentration and surface area for the substrate, accompanying with removal of large amounts of lignins and hemicelluloses. The present study demonstrated that one-step process based on successively hydrothermal and alkaline treatment could be considered as a promising technology to achieve the high concentration of glucose from wheat straw.

## Methods

### Raw materials

Wheat straw was harvested from farmland in Qishan county, Shaanxi province, China. They were first dried and then ground using a mill to obtain a 20–40 mesh powders. Then, the particle was extracted with toluene–ethanol (2:1, v/v) in a Soxhlet apparatus for 6 h to remove extractives and then dried to serve as control substrate.

### One-step process based on order of hydrothermal and alkaline treatment

The dewaxed powders were divided into four batches (each 7.0 g). Four processes were performed in a batch reactor (100 mL internal volume, Sen Long Instruments Company, Beijing, China), respectively. Specific processes were as follows: (1) 7.0 g powders were treated at 170 °C for 30 min under a solid-to-liquor ratio of 1:10 (g/mL); (2) 7.0 g powders were treated with 2% NaOH aqueous solution at 90 °C for 120 min under a solid-to-liquor ratio of 1:10 (g/mL); (3) 7.0 g powders were treated under the same alkaline treatment condition as stated above. Then, the reaction system was neutralized with 6 M HCl, followed by heating to 170 °C for 30 min; and (4) 7.0 g powders were treated under the same hydrothermal treatment condition as stated above. At the end of reactions, the reaction system was neutralized with 6 M NaOH, followed by cooled down to 90 °C and simultaneously added 2% NaOH for 120 min.

Aforementioned, the four one-step processes based on hydrothermal and alkaline treatment were named HP, AP, AHP, and HAP, respectively. After the reaction completed, the solids were separated through filtration with a Buchner funnel, washed thoroughly with distilled water, and then further dried in a cabinet oven with air circulation at 60 °C for 16 h. According to different processing conditions, the dried solids were named HR, AR, AHR, and HAR, respectively. However, the hemicelluloses and xylooligosaccharides in liquid were not recovered and analyzed due to the diversity of polymerization degree. During the biorefinery process, lignins were received due to their potential values in developing biobased materials and chemicals, which will be published shortly in another article. All of the prepared substrates (control, HR, AR, AHR, and HAR) were used to produce glucose and xylose by enzymatic saccharification in the present study.

### Enzymatic saccharification

The saccharification experiments were carried out at 2% of substrate (w/v) in 10 mL of 50 mM sodium acetate buffer (pH = 4.8) in a double-layer shaking incubators (ZWYR-2102C, Shanghai, China) at 50 °C for 72 h. The speed was performed at 150 rpm. Cellulase (15 FPU/g substrate), which was purchased from Novozymes North America, Inc. (Franklinton, NC), was used for all the saccharification experiments. The hydrolyzates were measured by a high-performance liquid chromatography (HPLC) system (Agilent 1200 series, Agilent Technologies, USA). The measurements were conducted in triplicate, and the relative standard deviation was found to be below 3.0%. The data represented are the averages obtained from experiments.

### Analysis methods

The chemical compositions (%, w/w) of the substrates were measured by the NREL standard analytical method [13]. Analyses of the carbohydrates of the substrates were assessed by HPLC under the same conditions as enzymatic hydrolysates. CP/MAS ^13^C-NMR spectra of the substrates were conducted using a Bruker AV-III 400 M spectrometer (Germany) [19]. XRD analysis of the substrates was recorded by a D/MAX 2500PC diffractometer (Rigaku Corporation, Japan). The crystallinity indexes (CrIs) of the substrates were determined from the ratio of the crystalline peak area to the total area of crystalline and amorphous peaks. SEM images of the substrates were performed with a Phenom XL (Phenom-World, Netherlands) instrument at 10 kV. BET surface areas of the substrates were measure by analyzing of the nitrogen adsorption using an SSA-7000 surface area analyzer (Beijing Bi’aode Electronic Technology Co., Ltd., Beijing, China) after 8 h of degassing at 120 °C and 1 h of degassing at 150 °C. The measurements (chemical compositions and BET surface areas) were conducted in triplicate, and the relative standard deviation was below 5.0%. The data represented are the averages obtained from experiments.

## Abbreviations

CP/MAS ^13^C NMR: cross-polarization/magic angle spinning ^13^C nuclear magnetic resonance
XRD: X-ray diffraction
SEM: scanning electron microscopy
BET: Brunauer–Emmett–Teller
HP: one-step process based on hydrothermal treatment alone
AP: one-step process based on alkaline treatment alone
AHP: one-step process based on successively alkaline and hydrothermal treatment
HAP: one-step process based on successively hydrothermal and alkaline treatment
HR: the residue obtained from hydrothermal treatment alone
AR: the residue obtained from alkaline treatment alone
AHR: the residue obtained from one-step process based on successively alkaline and hydrothermal treatment
HAR: the residue obtained from one-step process based on successively hydrothermal and alkaline treatment
NREL: National Renewable Energy Laboratory’s
CrIs: crystallinity indexes
HPLC: high-performance liquid chromatography
